# Exploring the perspectives and experiences of health workers at primary health facilities in Kenya following training

**DOI:** 10.1186/1752-4458-7-6

**Published:** 2013-02-04

**Authors:** Rachel Jenkins, Caleb Othieno, Stephen Okeyo, Julyan Aruwa, Jan Wallcraft, Ben Jenkins

**Affiliations:** 1 Epidemiology and Mental Health Policy, and WHO Collaborating Centre (Mental Health), Institute of Psychiatry, King’s College London, PO35, David Goldberg Centre, De Crespigny Park, London, SE5 8AF, UK; 2 Department of Psychiatry, University of Nairobi, Nairobi, Kenya; 3 Great Lakes University, Kisumu, Kenya; 4 Great Lakes University, Kisumu, Kenya; 5 University of Birmingham, Birmingham, UK; 6 Zacchaeus 2000 Trust, London, UK

## Abstract

**Background:**

A cluster randomised controlled trial (RCT) of a national Kenyan mental health primary care training programme demonstrated a significant impact for health workers on the health, disability and quality of life of their clients, despite a severe shortage of medicines in the clinics. In order to better understand the potential reasons for the improved outcomes in the intervention group, the experiences of the participating health workers were explored through qualitative focus group discussions, as focus group methodology has been found to be a useful method of obtaining a detailed understanding of client and health worker perspectives within health systems.

**Methods:**

Two ninety minute focus groups were conducted in Nyanza province, a poor agricultural region of Kenya, with 10 health workers from the intervention group clinics where staff had received the training programme, and 10 health workers from the control group where staff had not received the training during the earlier randomised controlled trial.

**Results:**

These focus group discussions suggest that the health workers in the intervention group perceived an increase in their communication, diagnostic and counselling skills, and that the clients in the intervention group noticed and appreciated these enhanced skills, while health workers and clients in the control group were both aware of the lack of these skills.

**Conclusion:**

Enhanced health worker skills conferred by the mental health training programme may be responsible for the significant improvement in outcome of patients in the intervention clinics found in the randomised controlled trial, despite the general shortage of medicines and other health system weaknesses. These findings suggest that strengthening mental health training for primary care staff is worthwhile even where health systems are not strong and where the medicine supply cannot be guaranteed.

**Trial registration:**

ISRCTN 53515024

## Background

Mental disorders are common in primary care across the world. A few specific interventions for single disorders or single client groups in low income countries have been evaluated but it remains challenging to scale up for all the usual mental disorders and client groups within existing human and financial resources [1,2]. Since 2005, the Kenya Ministry of Health has conducted a programme to train 3000 level 2 and level 3 primary health care staff across Kenya in collaboration with the Kenya Medical Training College (KMTC), Kenya Psychiatric Association and the WHO Collaborating Centre, Institute of Psychiatry, funded by Nuffield International Foundation and using a sustainable general health system approach [3,4].

In 2010 the research project conducted a phase 2 exploratory trial as a pilot cluster randomised controlled trial (RCT) testing the effect of an affordable low cost training intervention, which was integrated with the national health sector reforms. The training intervention was accompanied by the World Health Organization primary care guidelines adapted for Kenya, and was also intended to be accompanied by routine supervision from district staff and by routine availability of medicines in the clinics. The trial assessed the impact of the training programme: (i) on the competencies of primary care staff to recognise mental disorders, treat and make appropriate referrals to the scarce specialist service; (ii) on recovery (improved health and social outcomes and quality of life) of clients. The trial was conducted in a real clinical field setting, using local trainers (who had been trained in 2006, received a refresher course in 2009 and had delivered several such courses per year since 2005) to train health staff in the intervention group. The project did not exert any special influence on the usual local availability of medicines, district supervision or local health management information systems. During the course of the trial Kenya, and especially Nyanza Province, experienced a severe shortage of medicines, and this was reflected in the research findings on medicine availability in the clinics participating in the trial. Nonetheless, the trial found significant improvement in the clients of the trained health workers in the intervention group compared with those in the control group [5]. Since focus group methodology has been found to be an effective way to explore health worker and client views within health system contexts [6], and at the request of the funder (the UK Department for International Development), we therefore conducted focus groups with some health workers and clients from the trial to better understand their perspectives and experiences. This paper reports the experiences of some of the health workers in the trial.

## Methods

We contacted 20 health workers from 20 of the 100 clinics which had taken part in the RCT and invited them to take part in one of four ninety minute focus groups (10 primary care workers selected from the intervention group, and 10 primary care workers selected from the control group). We paid respondents’ transport and subsistence for the day they came for the interviews. No additional payments were made. There was no communication about the study between the different groups of participants before their respective focus groups were held.

### Ethical considerations, explanation and consent

An explanatory information sheet was given to each client and health worker in English and Kiswahili, and read out to those who could not read. We explained verbally in English, Kiswahili and Luo to the participants the purpose of the meeting and asked for their individual consents. They were reassured of confidentiality and told that we wanted to learn from their experiences. We then assigned each participant an identification number. They were told that the conversations would be taped but they would not be identified by name.

The focus group discussions were held in August 2011, eight months after the end of the randomised controlled trial, during a residential two days in a small hotel at Chulaimbo, near Kisumu, which was easily accessible by all those invited. They were conducted by CO assisted by RJ, and each lasted 90–120 minutes. Discussions were conducted mainly in English, but also in the local language where participants could not understand English or found it easier to express themselves in Luo. A voice recording of all the sessions was made, and both RJ and JA took written notes which were used to help clarify the transcript material where necessary.

### Instruments

The focus group discussions were guided by the following exploratory questions:

To start with, could you tell us what you know about mental health?

What are your views concerning psychiatric disorders in this district, and in this health centre?

What are the common types of mental/psychiatric disorders encountered locally?

What is your experience in managing these cases?

Could you describe a specific case?

Are there any agencies involved in the management of the mentally ill? Please give examples.

In what way do they help?

Have you had any training in mental health? Please specify.

Did you attend the recently concluded training for primary health workers? Please describe your experience.

What were the positive things about the training?

What were the negative things about the training?

Did you benefit from the training?

How has it affected your practice?

Have the patients benefited in any way? Please give a specific example.

Please describe how you apply the knowledge and skills learnt in the training? If it is not applicable, please explain why.

Would you recommend the training to a colleague?

### Data analysis

The recordings of the discussions were transcribed and translated into English where necessary. The voice recordings and the transcripts were analysed for common themes emerging in response to the guideline questions. Matrices were created to help facilitate the comparison of text across the different categories of informant.

## Results

11 health workers attended from the control group and 14 from the intervention group. This was more than the numbers invited, but these additional 5 attendees fulfilled eligibility criteria of having taken part in the RCT, and were therefore included in the respective focus groups, and their statements are included in this analysis. Some health workers who had not been part of the randomised controlled study also arrived at the focus group venue. They were not sent away as many had travelled far, and were therefore placed in a separate focus group for the purposes of discussion. As they had not received the training intervention, their comments are included here as part of the controls.

### Understanding of mental health and mental illness

The health workers in the intervention group were able to recognise the less severe as well as more severe forms of mental disorder and hence perceived the load of mental illness to be high in the community, and described a broad spectrum of mental health problems seen in the clinics

*“I feel that mental illness is very important because like in our facility we can see like two clients a week*. *Mostly they are psychotic*.” Health worker 5, intervention group.

In contrast, the health workers in the control group thought that mental illness occurred only “*once in a while*”. The health workers from the control group thought that mental illness occurred in only around 5% of the patients seen. They had a narrower concept of mental health compared to those in the intervention group.

“*At Kinasia dispensary*, *the number of cases that I have seen I can place them*

*at may be five percent*……*mostly they are epileptic*” Health worker 18 control group.

The same health worker from the control group could also relate mental illness to HIV cases but only mentioned those who had severe forms of mental illness – exhibiting overt disturbed behaviour such as increased talkativeness, disorientation or running away.

“*In the dispensary where I am working at those who have mental cases are those who do not accept that they are positive and if they are told to come for drugs they don*’*t want to come and when their CD4 count becomes low they start talking too much and some even want to run*. …….. *When their CD4 comes down they get mad and when talking to them they do not reason meaning that their mental status is not normal*. “Health worker 18 control group.

Interviewer: *So is that how you can tell when you think they are mad because they are not making sense*?

“*They keep on saying different things*. *If you ask him for example where they comes from maybe he comes from near a place like Gem*, *he will tell you that he comes from Obaga and not near their*”

Health worker 18 control group:

“*Mental illness sometimes you may notice that somebody may be partially unconscious*, *may be talking to himself or whenever you talk to him may be he doesn*’*t answer something like that*. *When they come to the clinic may be wherever he sits he talks to himself at least there is something disturbing his mind*. *So those are the signs you look for*. “Health worker 1 control group.

The health workers in the intervention group were able to articulate broader concepts of mental health problems presenting in the clinics.

“…. *apart from the HIV psychosis my colleagues are talking of*, *there are also issues of attempted suicide that we*’*ve had*. *Patients taking poison so upon his taking you find that there are social factors*, *issues with the family*, *disagreements in the family*, *joblessness*, *depression*……… *sometimes they have delusion type of state so many a times it revolves round the socio*-*economic factors apart from the HIV psychosis when now they become sick*. …….. *even those who take poison*…. *the suicide attempts*… *when you take history and you find even more why they took poison there is something to do with the psychology*, *something somehow was not right*, *the person get depressed and opt to terminate his or her life so those are the sorts of mental cases that we see*. *Suicide attempts*, *the HIV psychosis*, *depression is like in all patients mostly those with terminal illnesses but the degree now matters*.” Health worker 15 ..intervention group.

The intervention group displayed broader concepts of causation of mental illness than those in the control group, were aware of the concept of vulnerability and recognised that anyone could have mental health problems:

“*I want to say that the training really helped me to at least see better ways of helping this mother*. *Yes*, *because I realised the mother had got dual areas of stressful life*. *Not having a child of her own and now dedicated so much on the husband who has also now deserted her*. *So you could see the situation*.” Health worker 20, intervention group.

Examples of cases described by the health workers in the intervention group included mothers who are stressed:

“*There are cases where mothers are breadwinners* - *their husbands do not take care of the families so they are stressed most of the time*.” Health worker 12, intervention group.

Infertility in females:

“*Mothers who have not given birth for a long time may get stressed up because of their husbands and mother*- *in*-*law*.” Health worker 22, intervention group.

Relational problems between couples:

“*Mothers who come into the health facilities and you find that the husband has gotten another wife*, *now this first one is fully neglected*”. Health worker 23, intervention group.

“*A woman who had never given birth*, *in a polygamous relationship* …*so you see this is a woman who has been going to several health facilities with several complaints*. *She has been tested for typhoid*, *malaria* … *so every two weeks she comes with features of malaria*, *and you see I had to go through the process once again during the assessment and eventually I realised this lady had got some stressful life*.” Health worker 20, intervention group.

Cases of male impotence:

“*If you listen to FM radio*, *you realise there is a lot of advertisement in impotence*, *people who are treating impotence*, *and you realise it is more common* … *it is called* “*Oae pacho*” (*the person has left the home*) *or* “*uacha*” (*nothing in the trousers*).” Health worker 20, intervention group.

And drug abuse:

“*With the current trends of life in Kenya*, *inflation and poverty*, *you may find many young men who indulge in drug abuse whereby they take* “*bhangi*” (*cannabis*) *and in the long run they develop features like psychosis*.” Health worker 25, intervention group.

The control group understood mental illness as either psychosis or as HIV related emotional conflict:

“*Especially those who have been HIV infected because you know it depends on how somebody is infected*. *Like in a couple*, *you find the discordant couples whereby they will have problems*. ‘*You are the one who brought it*, *how come I am negative and you are positive*?’ *It means there will be mental torture in the home*”. (Health worker 1 control group)

Health workers in the control group realized that they often neglected psychological issues

*Also to add on that mental health is very important because many a times we have dealt with the physical part of the patients or rather the clients we meet forgetting their psychological problems*. *Like a patient could come and you are just interested in taking their temperature as physical illnesses while the real idea is in the mind so you see even if you don*’*t address issues of the mind even prognosis of the illness the rate at which one will get better is also slowed because many a times you will imagine its malaria when the issue is not even malaria it could be other form of social factors at home or some other things so mental health must be addressed it is scored in the community and everywhere we work*. (Health worker 6 control group)

Child abuse was raised by both groups as a serious and widespread problem:

“*Nowadays you can see many cases of children being abused*; *not only the girl child but also boys*, *they are molested*.” (Health worker ID missing data)

Both intervention and control health workers argued for more education to the community about mental illness:

“*It will help so much before even the clients reach the hospital*. *Because they are seen as people who are stubborn*, *they are tied up with ropes*, *they are tied up with chains and then they are beaten up which is just because they do not understand*. *They have little knowledge about mental illness*.” Health worker 6, control group.

### Effect of the training programme on health worker practice

#### Educational talks for clients

The health workers who had received the training reported that they had now included mental health in the educational talks which they routinely give each week in their clinics:

“*What I can say is that when you are having these health talks in the morning with the clients who come*, *you can be able to give them information about health issue especially with the mental health so that they can become aware*, *and if at all they have any people who have this mental problem*, *they can bring them to the hospital or to the dispensaries*.”

#### Holistic bio-psycho-social assessment and management

They were now able to appreciate the morbidity due to mental illness and to adopt a more holistic approach in the assessment and management of patients. They were now better able to manage the patient, involve the patient and relatives in the management, and refer those that needed specialised treatment. They said they were less likely to miss mental disorder accompanying physical complaints and conditions:

“*It was a very good eye opener and we realised that we have not been managing the clients with mental illnesses* … *and we also realised that other mental illnesses could manifest in other medical conditions*.” Health worker 20, intervention group.

“*I want to say I have gained an experience to give a holistic approach to the assessment*. *Let*’*s say when a client comes*, *you don*’*t just think of the client in terms of the physical illness*. *I also look at the client in all other ways*, *something that I was not doing sometimes back*.” Health worker 20, intervention group.

“*I realised that people used to misdiagnose most cases* … *and waste a lot of anti*-*malarials*.” Health worker 19, intervention group.

“*I want to say that the training really helped me to at least see better ways of helping this mother*. *Yes*, *because I realised the mother had got dual areas of stressful life*. *Not having a child of her own and now dedicated so much on the husband who has also now deserted her*. *So you could see the situation*.” Health worker, 20, intervention group.

The health workers from the control group were acutely of their deficiencies when it came to managing emotional problems of their clients. They had difficulties in exploring and eliciting the root causes of the patients’ problems. This led to unsatisfactory outcomes as illustrated below.

“*here is this patient who came with her baby to seek medical attention and said she had* “*high blood pressure*”. *When her pressure was about to be checked*, *she started sweating and shaking*. *When I asked her why she was shaking she told me that it is because of the pressure*. *I talked to her and explained to her that I also suffer from the same and the best thing is to relax so that her pressure can be checked which she agreed*. *After her blood pressure was checked she sat on the same seat from morning to evening*. *When I ask her why she was not going home she told me that if she go home she will die*. *I talked to the patient and took her to my house to spend the night there since my house was in the hospital neighborhood*. *The husband came in the morning and he was very wild and the woman was also crying saying that it is the husband who infected her*. *So I was just wondering if such a thing happens and one of the partners come to the hospital alone and there is need to give him*/*her counseling what can one do*?” Health worker 19 control group.

The control health workers described particular difficulties talking to spouses of HIV positive clients.

“*I wonder where to start from before talking to male clients especially when it comes to counseling them in cases of HIV* “

Another health worker from the control group expressed her fears

“*It is a problem because you might break that family*”. Health worker 14 control group.

The control health workers appreciated that they did not have the core skills necessary to do basic management of mental health problems

“*And also health workers we are limited of skills to handle this issue*. *We should be also be taught how to manage stress and also manage mental health problems*.” Health worker 4 control group

And that this lack of skills resulted in unnecessary referrals to secondary or tertiary care when in fact the problems could be handled locally.

“*I lack skills because when I see a mental ill person am like the patient should go toward eight in Kisumu or in Mathari* (*Referral Hospital in Nairobi*) *when we can also set up our units and manage these people at our level*. “Health worker 6 control group

#### Thorough history taking

Further the intervention group realised that thorough history taking was essential, that not all somatic complaints are due to physical illnesses, and thus to not wrongly attribute such cases to malaria:

“*Well if you take a good history somebody will tell you that* ‘*previously I have been undergoing this treatment of malaria and do not get on well*’, *so at least with good counselling and history taking you will realise that there is something missing here*.” Health worker 13, intervention group.

“*Without proper history taking you can be given the wrong drugs*, *and also drugs can harm you in another way medically*. *So there are some people who do not take good history*. *They will treat you for malaria or any other things while you don*’*t suffer from that and that one can bring you complications*. *You see a case where somebody was treated with the steroid drugs and he got permanent damage of the kidney while the person was suffering from a mental illness*. *It can be worse if not diagnosed properly*.” Health worker 26, intervention group.

“*The importance of taking proper history*.” Health worker 22, intervention group.

“*I have two school girls* … *who presented with convulsion disorder and chest pains*. *I examined them* - *you find they have no problem*. *And when you take history*, *you find they have stress at home*.” Health worker 21, intervention group.

They spent more time with the patients trying to understand and solve their problems:

“*After training we had an opportunity of making a consultation room into a* ‘*talking ward*’, *so* … *when a person bring a client to the dispensary*, *they can say* ‘*Sister*, *I have got somebody who looks like Amugoroki*’ - *that is someone who is somehow possessed*. *So we are encouraging them*. *So you can find at least maybe they are having some conditions which we were taught*. *So talking works*!” Health worker 12, intervention group.

“*Yes in my case I have been able to create an insight with clients using my counselling skills* … *I have been able to make them open up by talking about the cause of a problem*.” Health worker, intervention group.

Another commented that the patients had benefitted from the training given to the health workers because:

“*Like long time ago*, *patients used to come to the facility with fixed mind*. *They wanted valium injection*, *but with counselling method*, *telling them or even having dialogue with them at least they know that everything is not only injection*. *So they have got change of attitude also*.” Health worker, intervention group.

One participant said that the training made him aware that stress could be a cause of non-compliance among HIV patients attending comprehensive care units and accordingly took time to follow up those who missed their appointments:

“*Maybe in addition to that it has really helped us*. *Maybe you have put a client in to come on such and such a date*, *and that client fails to come*. *We normally concentrate on adherence counselling but currently we have jotted in maybe the mental health also because maybe such a person might be having stress also for him or her to fail to come for that return date*.” Health worker 16, intervention group.

#### Involvement of the community

This was crucial and some of the trained health workers had opened dialogue with the community members through health education talks given in the community meetings (Chief’s “baraza” and church gatherings) which, in their view, led to greater acceptance of the patients in the community and helped to de-stigmatise mental illness:

“*After the training*, *what me and my colleague did*, *first we decided to go to the chief barazas and informed them that even mental illness would be treated at our local dispensary*, *and then we approached the in*-*charge of the Siaya district psychiatric unit who promised to give us some drugs*, *so that as we get the clients we would forward their names and could come on a weekly basis*, *see them but leave us with some drugs to be giving them every month*.” Health worker, intervention group.

The trained health workers recognised that the reintegration of mentally ill patients with other types of patients made them feel accepted by the general community, enhanced recovery and prevented psychosocial exclusion. Some health workers said they had been able to form patient support groups but that it was challenging since the patients were not homogeneous and had differing needs:

“*And unlike in HIV where we really are able to form support groups*, *mental illness is a bit very challenging to form a support group or same because some people have got diverse*, *mental illnesses are diverse because traditions are completely very different*. *So when you want to put them together*, *it will be like who are we*, *who are these people*.” Health worker 20, intervention group.

#### Rehabilitation

The health workers however had not so far been able to help patients with specific activities of daily living, or help with workplace skills, but they did feel that the possibility of assessment and treatment at primary care level was better for speedy rehabilitation than admission to a level 4 hospital:

“*Yes*, *I think it has done a lot to rehabilitate these patients because*, *let*’*s say when they are taken to a psychiatric unit*, *some of them don*’*t get well quickly enough because there is that mentality that the patient feels*, *he is mentally ill*. *And as the integration to these other health centres*, *the patient just feels as if he*’*s among other clients who are sick maybe with malaria or these other types of illnesses*, *so psychologically he feels he is being managed as a patient with malaria*, *in a way it makes him recover very fast*.” Health worker, intervention group.

“*I think it has improved our referral system because whenever we have patient who is not responding to treatment*, *we follow the district psychiatric nurse to come and review*.” Health worker 26, intervention group.

## Discussion

This is the first qualitative focus group study of health workers involved in a randomised controlled trial of primary care training on mental health in Kenya. The study was conducted in Nyanza province, Kenya, in the public sector primary care system, and the study findings are limited to this group, although are likely to have wider relevance for Kenyan primary care as a whole. Further limitations of the study include the fact that most of the research team for the focus group study were also involved in the randomised controlled trial. The use of both clients and health workers affords some triangulation of the findings (see accompanying papers [4,5]).

From the focus group discussions it seems that trained health workers reported being more effective than the untrained health workers in assessment, diagnosis and treatment. The skills learned during the training seemed to help, not only in the better management of psychosocial problems, but also in better management of physical disorders such as HIV, by appropriate counselling and decreasing non-compliance with medications.

Since the drug supply was poor in both the control and the intervention groups it is likely that these enhanced psychosocial skills in the health workers, noticed by both themselves and their clients (see accompanying papers [4,5]), are the factors that contributed to better recovery in the clients in the intervention group compared to those in the control clinics in the randomised controlled trial reported elsewhere [5].

## Conclusion

These focus group discussions suggest that the health workers in the intervention group perceived an increase in communication, diagnostic and counselling skills, while health workers in the control group were aware of the lack of these skills. These findings are triangulated to some extent by the findings in the accompanying paper about client experiences [5], where the clients in the intervention group noticed and appreciated enhanced communication, diagnostic and counselling skills in their respective health workers, whereas clients in the control group were aware of the lack of these skills. These enhanced generic health worker skills conferred by the mental health training programme may be at least partly responsible for the significant improvement in outcome of patients in the intervention clinics where staff had received the training programme, compared to the control group, despite the general shortage of medicines and other health system weaknesses.

These study findings concur with those found in a randomised controlled trial of the same training programme in Iraq, where research observers found better diagnosis and communication skills in staff trained in the program, compared to controls [6].

These findings support the value of strengthening mental health training for primary care staff and indicate that such training is worthwhile even where health systems are not strong and where the medicine supply cannot be guaranteed.

## Competing interests

The authors declare that they have no competing interests.

## Authors’ contributions

RJ was responsible for overall design of the study, obtained funding, and co-led the focus groups with CO, wrote the first drafts of the introduction and discussion, and subsequent drafts of the overall paper. CO chaired the focus group discussions, supplied the transcription of data and the main analysis of findings, and wrote the first drafts of the methods and results. SO supervised the local implementation of the study design. JA coordinated the invitations of client and health workers, and took notes of the focus group discussions. JW checked the qualitative methodology and contributed to UK comparisons of the discussion. BJ advised on the specific content of the focus groups, and organised the audio taping of the discussions. All authors contributed to and approved the final version of the paper.
